# Analysis of Mesoscopic Structured 2-Propanol/Water Mixtures Using Pressure Perturbation Calorimetry and Molecular Dynamic Simulation

**DOI:** 10.1007/s10953-016-0554-y

**Published:** 2016-12-17

**Authors:** Jordan W. Bye, Colin L. Freeman, John D. Howard, Gregor Herz, James McGregor, Robert J. Falconer

**Affiliations:** 1 Department of Chemical & Biological Engineering, ChELSI Institute, University of Sheffield, Sheffield, S1 3JD England, UK; 2 Department of Material Science & Engineering, University of Sheffield, Sheffield, S1 3JD England, UK

**Keywords:** Isopropanol, Differential scanning calorimetry, Heat capacity, Thermal expansion coefficient, Molar expansivity

## Abstract

In this paper we demonstrate the application of pressure perturbation calorimetry (PPC) to the characterization of 2-propanol/water mixtures. PPC of different 2-propanol/water mixtures provides two useful measurements: (i) the change in heat (Δ*Q*); and (ii) the $\left[ {\delta \bar{C}_{p} /\delta p} \right]_{T}$ value. The results demonstrate that the Δ*Q* values of the mixtures deviate from that expected for a random mixture, with a maximum at ~20–25 mol% 2-propanol. This coincides with the concentration at which molecular dynamics (MD) simulations show a maximum deviation from random distribution, and also the point at which alcohol–alcohol hydrogen bonds become dominant over alcohol–water hydrogen bonds. Furthermore, the $\left[ {\delta \bar{C}_{p} /\delta p} \right]_{T}$ value showed transitions at 2.5 mol% 2-propanol and at approximately 14 mol% 2-propanol. Below 2.5 mol% 2-propanol the values of $\left[ {\delta \bar{C}_{p} /\delta p} \right]_{T}$ are negative; this is indicative of the presence of isolated 2-propanol molecules surrounded by water molecules. Above 2.5 mol% 2-propanol $\left[ {\delta \bar{C}_{p} /\delta p} \right]_{T}$ rises, reaching a maximum at ~14 mol% corresponding to a point where mixed alcohol–water networks are thought to dominate. The values and trends identified by PPC show excellent agreement not only with those obtained from MD simulations but also with results in the literature derived using viscometry, THz spectroscopy, NMR and neutron diffraction.

**Electronic supplementary material:**

The online version of this article (doi:10.1007/s10953-016-0554-y) contains supplementary material, which is available to authorized users.

## Introduction

Pressure perturbation calorimetry (PPC) is the measurement of pressure induced heat changes in a sample. Identical pressure pulses are applied to a reference and a sample cell, and the difference change in heat (Δ*Q*) between the two cells is measured using a calorimeter. This technique can be applied across a range of temperatures. Early PPC subjected the samples to high-pressure changes (up to 4000 bar) and was suitable for studying phase transitions in organic solvents and polymers [1–4]. The current low pressure, high sensitivity, PPC technology became available around 2001 [5]. A modification to existing highly sensitive differential scanning calorimetry (DSC) instrumentation has enabled this type of analysis to be carried out at lower pressure changes (± 4 bar). There are relatively few studies on small molecules in water using PPC analysis [6–8]. The majority of published studies investigate macromolecules including synthetic polymers [5, 9–11], proteins [12–17] and nucleic acids [18, 19] in pure water or in buffered solutions. The results are expressed as the calculated thermal expansion coefficient (*α*). Research into lipids in water has employed PPC to investigate structures such as micelles and lipid bilayers with the results often expressed in terms of molar expansivity (*E*) [20–23].

In this paper we describe the application of PPC and molecular dynamics (MD) simulation to study 2-propanol/water mixtures. Understanding the structural dynamics of the binary 2-propanol/water systems at the molecular scale is of interest as these mixtures may be present in products such as disinfectants, paint and ink formulations, fuel additives and deicer products. 2-Propanol/water mixtures were selected in this study as they have previously been widely investigated using a variety of analytical technologies including viscosity measurements [24], neutron diffraction [25], nuclear magnetic resonance spectroscopy (NMR) [25, 26] and terahertz time domain spectroscopy (THz-TDS) [25, 26]. The non-ideality of 2-propanol water mixtures has also been demonstrated and explanations for the observed non-ideality proposed. This system therefore provided an ideal mixture to demonstrate the utility of PPC and to enhance our ability to interpret PPC data.

The goal of the research presented in this paper is to demonstrate the potential of PPC analysis as a tool to investigate the behavior of aqueous solutions of small molecules such as 2-propanol through measuring the difference in heat (Δ*Q*) and the gradient $\left[ {\delta \bar{C}_{p} /\delta p} \right]_{T}.$ Complementary MD simulations were employed in order to correlate the observed PPC results with the mesoscale structure of the solutions. These were further compared and contrasted with previous literature studies employing a variety of methods including viscosity measurements, neutron diffraction, NMR spectroscopy and THz-TDS analysis [24–26]. PPC is a relatively quick, easy-to-use, technique that enables direct measurement of thermodynamic parameters which represents a valuable addition to the analytical toolkit in this field.

## Molecular Dynamics Simulations

Simulations were performed using DLPOLY classic [27] with a time step of 0.5 fs. All the simulations were carried out at a temperature of 25 °C and a pressure of 1 bar with a NPT ensemble using a Nose–Hoover thermostat and barostat with relaxation times of 0.01 and 0.05 fs.

Simulations were run on configurations with a total of 3000 molecules with mole percentages of 2-propanol of 0, 1, 2.5, 5, 10, 15, 20, 25, 30, 35, 40 and 100%. The simulation box was generated by randomly mixing the appropriate number of water and 2-propanol molecules using the packmol software [28] with a tolerance of 2.0. Simulations were then equilibrated and then run for several ns until convergence of the configurational energy and enthalpy was reached. Convergence was judged to have occurred when the configurational energy of four successive block averages of 250 ps were within 50 kJ·mol^−1^ of each other. The water molecules were modelled using the TIP3P [29] forcefield and the 2-propanol molecules were modelled with the general amber forcefield [30], specifically the gaff03 forcefield. Charges for 2-propanol were calculated using the semi-empirical method AM1-BCC [31] and standard Lorentz–Berthelot mixing rules were used for the cross term interactions between the 2-propanol and water molecules. The short-range interactions of the forcefields were cutoff at 10 Å.

The hydrogen bonding of the mixtures was calculated from the final 1 ns of the MD simulations. A hydrogen bond was registered if the angle between the H–O–H was between 150° and 210° and the separation between the non-bonded oxygen atom and hydrogen atom was less than 2.5 Å.

The segregation of the water and 2-propanol molecules was examined and compared to random distributions of the 2-propanol and water molecules. This deviation from random distribution of the solution was examined by generating an ensemble of 2500 random arrangements of water and 2-propanol molecules with the same mole percentages as the MD runs with a box volume equal to that of the final MD simulations. Each of these configurations was then divided into 1000 equal sized smaller boxes and the number of water molecules and 2-propanol molecules appearing in each box recorded. Water molecules were defined to be present in a box if the oxygen atom was present and 2-propanol molecules were defined as present in a box if a carbon atom was present. For each water–propanol mix the count was then averaged across all 1000 boxes and normalized to give the fraction of boxes with a particular number of water and 2-propanol molecules. This generated a statistical distribution of boxes with the amount of water and/or alcohol molecules present. The same procedure was then carried out on the data from the MD simulations by using the trajectory of the final 1 ns of data with 2500 configurations for each simulation as outputted along the trajectory, ensuring the ensembles were the same size. The sum of the differences between the two distributions (random/ideal and real) was then recorded as the degree of non-ideality in the actual MD simulations via Eq. 1:

1 $$ {\text{Non ideality}} = \sqrt {\mathop \sum \nolimits [N_{\text{s}} \left( {x_{\text{s}} } \right) - N_{\text{R}} (x_{\text{R}} )]^{2} } $$

where the subscript s refers to the simulation data, the subscript R refers to the random distribution and *N*(*x*) is the fraction of the distribution with *x* alcohols present.

## Pressure Perturbation Calorimetry (PPC) Measurements

Ultra-pure water and 2-propanol were sourced from Sigma-Aldrich, Gillingham, UK with purities > 99.9%. PPC measurements were obtained using a capillary Nano-DSC (TA Instruments, New Castle, DE, USA). Samples were degassed for 20 min at 4 °C by vacuum to remove dissolved gas from samples and eliminate bubble formation during the scan. Heat changes (Δ*Q*) were measured during alternating pressure pulses of ± 4 bar from 1 bar to 5 bar at 1 °C intervals, every 10 min, from 7 to 62 °C, giving a usable data range of 9–61 °C. A heating rate of 0.1 °C·min^−1^ was used to satisfy isothermal conditions required during pressure pulses; this scanning rate is slower than the instrument feedback [19]. The instrument was held at a constant temperature for an hour before each scan to ensure that any asymmetry between the reference and sample cells was minimal. The Δ*Q* values were calculated by integrating the area underneath the thermal spikes that were caused by the pressure change, calculated using NanoAnalyze software (TA Instruments, New Castle, DE, USA) provided by the manufacturer. Water baseline scans were performed with pure water in both the reference and sample cell, while scans with 2-propanol present were performed with ultra-pure water in the reference cell and the water/alcohol mixture in the sample cell. The area under each thermal power spike was calculated by integration using NanoAnalyze software (TA Instruments, New Castle, DE, USA) and defined the heat change during pressurization for that temperature.

## Interpretation of the Δ*Q* Values

The Δ*Q* value of the 2-propanol water mixtures is, by the nature of the experiment, relative to pure water and is dominated by the energy released from the hydrogen bonds broken during pressurization.

The equations for analysis of PPC data were first derived and published by Lin et al. in 2002 [6], which was built upon the earlier work of Kujawa and Winnik in (see Supplementary Information) [5], i.e.:

2 $$ \Updelta Q = T\Updelta p\left( {\alpha_{0} - \bar{\alpha }} \right)V_{\text{part}} g_{\text{s}} $$

where $\bar{\alpha }$ is the thermal expansion of the solute partial volume, *α*_0_ is the thermal expansion of the solvent volume, *g*_s_ is the total weight of the solute and *V*_part_ is the partial specific volume of the solute. It was previously argued that while *g*_s_ *V*_part_ is not constant it can be treated as such, as *g*_s_ is decreased by displacement effects as temperature increases, while the *V*_part_ increases during heating [6]. The calculation of $\bar{\alpha }$ using Eq. 2 assumes that *V*_part_ is constant irrespective of concentration and this equation has been used extensively for the analysis of macromolecules in solution [6, 9–12, 15–19]. While this analysis may be valid for macromolecules, it has been shown that it does not hold for small solutes like salts [7] and it is therefore questionable as to whether it can be applied to 2-propanol–water mixtures. Additionally, this analysis only holds at a low solute concentration where the apparent volume of the solute approximates the partial specific volume at infinite dilution [8], and hence is not applicable over the entire concentration range.

For the study of ion pairs in solution, Eq. 3 was developed where the assumption is that ions have discrete hydration layers that can be treated separately from the unperturbed bulk water (see Supplementary Information) [7]:

3 $$ \Updelta Q = T{{\Updelta }}px_{\text{s}} \left[ {\left( {n + 1} \right)\left( {{\text{V}}_{\text{b}} \alpha_{\text{b}} } \right) - n\bar{V}_{\text{h}} \bar{\alpha }_{\text{h}} - V_{\text{s }} \alpha_{\text{s}} } \right] + A^{{\prime }} $$

where *V*_b_ is the molar volume of bulk solvent, $\bar{V}_{\text{h}}$ is the average molar volume of the solvent within the hydration layer; *V*_s_ is the molar volume of solute, *n* is the number water molecules in the hydration layer of the solute, *x*_b_ is the molar fraction of the bulk solvent, *x*_h_ is the molar fraction of the solvent within the hydration layer, *x*_s_ is the molar fraction of the solute, and *α*_b_, *α*_h_ and *α*_s_ are the thermal expansion coefficients of the bulk water, hydration layer and solute, respectively. The physical origin of *A*’ has not been established but may be a function of ion pair interactions.

For a 2-propanol water mixture, Eq. 3 can be applied at low 2-propanol concentrations where 2-propanol molecules exist as isolated species within a bulk water phase (taken as below 2.5 mol% in this work, see the discussion below). However, if 2-propanol molecules form a component of extended mesoscale networks, then Eq. 3 does not apply. Elsewhere, it has been suggested that methanol and water form separate, bi-percolating liquid networks at the concentrations where thermodynamic properties are at their maxima [32].

By the nature of the PPC measurement, the measured Δ*Q* value is related to the structural rearrangement of the solution upon the application of a pressure pulse. As the structure of alcohol–water mixtures is well-established to be dominated by hydrogen-bonding interactions, it is proposed herein that Δ*Q* be interpreted as the energy released from the hydrogen bonds broken during pressurization. As such, it is directly related to both the population of hydrogen bonds, i.e. between water and water, water and 2-propanol, and 2-propanol and 2-propanol, and also to the change in enthalpy associated with the breaking of these bonds. The absence or reduction of hydrogen bonding in the hydration layer around 2-propanol alkyl groups, or where the water is excluded by 2-propanol cluster formation, is therefore evident in the Δ*Q* value. The greater the extent of this reduction in hydrogen bonding then the greater the magnitude of the negative Δ*Q* value. Δ*Q* would hence be expected to decrease with increasing 2-propanol concentration.

## Interpretation of the $\left[ {\delta \bar{C}_{p} /\delta p} \right]_{T}$ Values

Interpretation of the physical origin of the $\left[ {\delta \bar{C}_{p} /\delta p} \right]_{T}$ value has been used to attempt to understand the relationship between water and solutes [8, 33]. In 1969 Loren Hepler used Eq. 4 to define the effect of a solute on water structure [33]:

4 $$ \left[ {\delta \bar{C}_{{p,{\text{part}}}} /\delta p} \right]_{T} = - T\left[ {\delta^{2} (V_{\text{part}} )/\delta T^{2} } \right]_{p} $$

where $\bar{C}_{{p,{\text{part}}}}$ is the partial molar heat capacity. Water was viewed as a mixture of two species: a bulky “ice-like” species with relatively low density and high structure, the other a denser less structured species. As temperature increases the proportion of the “ice-like” species was believed to decline, being replaced by the denser species. The characteristics of various solutes were interpreted in terms of their “structure-making” or “structure-breaking” capacity with alcohols being structure-making and electrolytes being structure-breaking. A similar logic was employed by Pielak and co-workers in 2004 in order to disprove the hypothesis that the “structure-making” or “structure-breaking” capacity of solutes was the origin of the Hofmeister effect [8]. They also substituted the definition for the thermal expansion coefficient ($\alpha = 1/V_{\text{part}} (\delta (V_{\text{part}} \bar{\alpha })/\delta T_{p}$) to give Eq. 5 [8]:

5 $$ \left[ {\delta \bar{C}_{{p,{\text{part}}}} /\delta p} \right]_{T} = - T\left[ {\delta \left( {V_{\text{part}} \bar{\alpha }} \right)/\delta T} \right]_{p} $$

Extending this logic, it is proposed herein that the $\left[ {\delta \bar{C}_{p,\text{part}} /\delta p} \right]_{T}$ value is not a measure of the “structure-making” or “structure-breaking” capacity of a solute, but instead can, at low solute concentrations, be due to the water in hydration layers around the solute molecules. Pielak and co-workers showed that hydrophobic solutes (1,3-dimethylurea, trimethylamine *N*-oxide dihydrate, 1,3-diethylurea) produced negative $\left[ {\delta \bar{C}_{{p,{\text{part}}}} /\delta p} \right]_{T}$ values; neutral polar solutes (sarcosine, urea, glucose, trehalose, sucrose, betaine, glycerol, stachyose, melezitose) produced weakly positive $\left[ {\delta \bar{C}_{{p,{\text{part}}}} /\delta p} \right]_{T}$ values; charged salts produced stronger positive $\left[ {\delta \bar{C}_{{p,{\text{part}}}} /\delta p} \right]_{T}$ values [8]. The higher charge density ion pairs {(NH_4_)_2_SO_4_, NH_4_Cl} produced higher positive $\left[ {\delta \bar{C}_{{p,{\text{part}}}} /\delta p} \right]_{T}$ values than low charge density ion pairs (guanidinium chloride, guanidinium thiocyanate). Later work suggested the $\left[ {\delta \bar{C}_{{p,{\text{part}}}} /\delta p} \right]_{T}$ value of the salt was related to the charge of the ion, rather than the charge density as had previously been proposed [7], the $\delta \left( {V_{\text{part}} \bar{\alpha }} \right)/\delta T$ value being positive for a hydration layer interacting with hydrophilic molecules and being negative for hydration layers interacting with hydrophobic molecules. This interpretation cannot be directly extended to higher solute concentrations, where complexes such as networks or micelles form, as the *V*_part_ value is determined at infinite dilution and assumes no inter-solute interaction. The interpretation of $\left[ {\delta \bar{C}_{p} /\delta p} \right]_{T}$ at such compositions remains open to conjecture. In the case of the PPC measurements used in this paper (where networks of 2-propanol in water are expected [25, 26]), the average molar heat capacity ($\bar{C}_{p}$) for the mixture is determined, avoiding the assumption of no inter-solute interaction when using the $\bar{C}_{{p,{\text{part}}}}$ value.

## Results

**Fig. 1:**
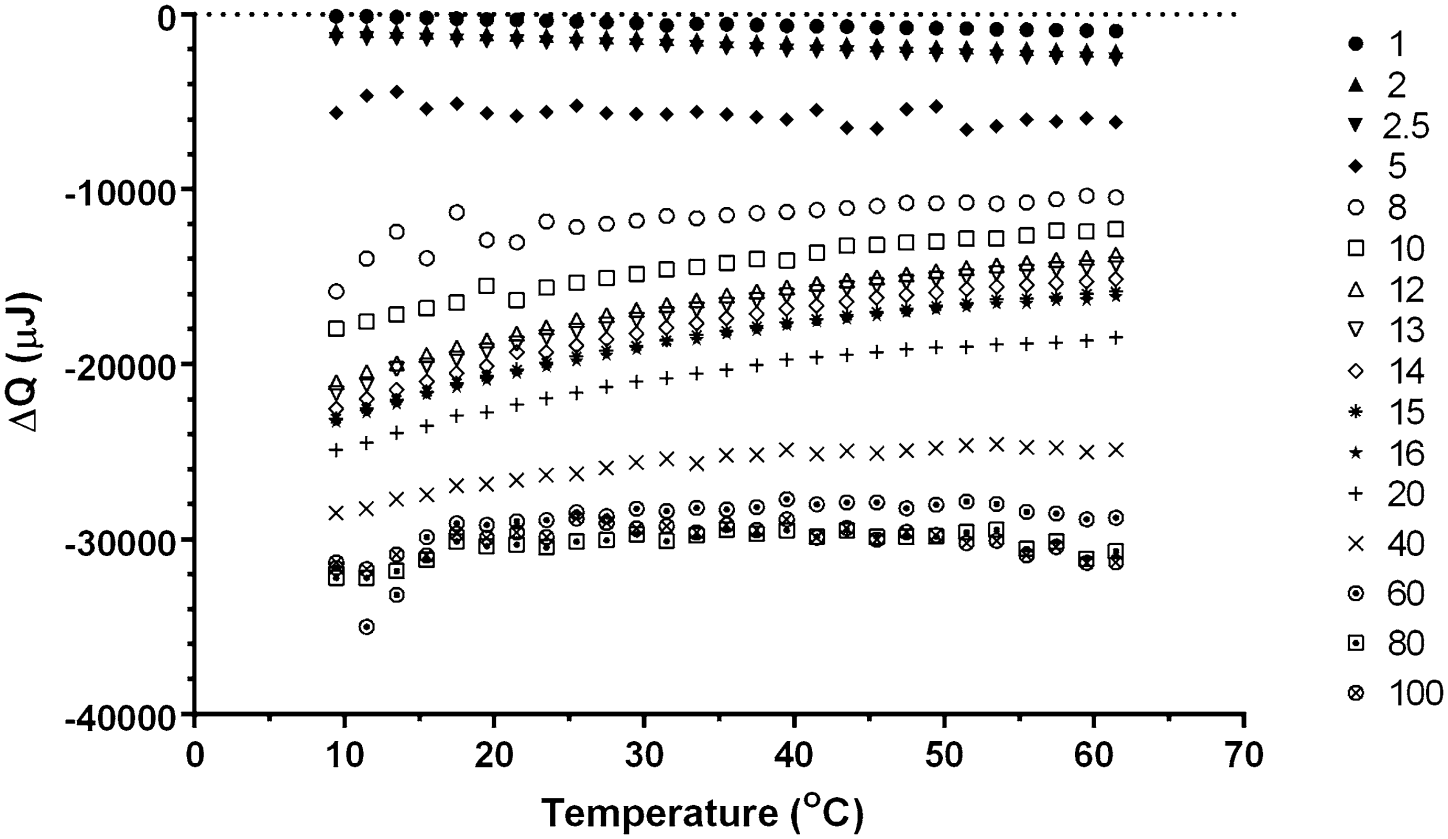
Heat changes from pressure increase (1–5 bar) from 7 to 65 °C for different 2-propanol mixtures. The 2-propanol composition from the top to the bottom curves is 1, 2, 2.5, 5, 8, 10, 12, 13, 14, 15, 16, 20, 40, 60, 80 and 100 mol%. Note the noisy data is at 5 and 8 mol% where the 2-propanol concentrations are higher than 40 mol%

The raw data from a pressure perturbation scan of 2.5, 5 and 15 mol% 2-propanol in water are shown in Figs. S1–S3 in Supplementary information. Alternating pressure pulses from 1 to 5 bar and then 5 to 1 bar were applied to the samples at 1 °C intervals from 7 to 56 °C, with a heating rate of 0.1 °C·min^−1^. The heat changes (Δ*Q*) recorded upon pressurization of 0–100 mol% 2-propanol/water mixtures at temperatures between 7–61 °C are shown in Fig. 1 and Table 1. Regardless of temperature, the Δ*Q* values become increasingly negative as the 2-propanol concentration increases; however different trends are observed as a function of temperature for the different compositions. Specifically, a negative gradient (Δ*Q* vs. *T*) is observed for concentrations between 1 and 2.5 mol% 2-propanol, while above 5 mol% a positive gradient is observed with the maximum gradient occurring at 14 ± 2 mol% 2-propanol (Fig. 1).

**Table 1:** The experimentally determined difference in heat (Δ*Q*) at 25.5 °C, the average gradient $\left[ {\delta \bar{C}_{p} /\delta p} \right]_{T}$ between 9 and 35 °C, $\left[ {\delta \bar{C}_{p} /\delta p} \right]_{T}$ between 35 and 61 °C, and the kinematic viscosity (*v*) at 25.0 °C at different concentrations of 2-propanol

Concentration of 2-propanol (mol%)	Δ*Q* (μJ)	$\left[ {\delta \bar{C}_{p} /\delta p} \right]_{T}$ 9–35 °C (mJ·bar^−1^·K^−1^)	$\left[ {\delta \bar{C}_{p} /\delta p} \right]_{T}$ 35–61 °C (mJ·bar^−1^·K^−1^)	*v* ^a^ (10^6^ m·s^2^)
0.0	70			0.89
1.0	−403	−4.8	−3.5	
1.5	−737	−5.1	−4.8	
2.0	−1243	−5.6	−5.6	
2.5	−1683	−5.6	−6.0	
5.0	−5214	−2.2	−3.0	
8.0	−12156	20.3	9.8	
10.0	−15324	34.1	18.4	2.59
12.0	−17469	45.3	22.0	
13.0	−18080	47.6	21.4	
14.0	−18923	49.1	21.1	
15.0	−19560	47.6	21.3	
16.0	−19785	47.8	20.0	
20.0	−21630	44.1	15.8	3.31
40.0	−26242	30.7	3.6	3.46
60.0	−28436	17.5	−6.7	3.02
80.0	−30103	13.9	−11.2	2.70
100.0	−28808		−20.4	2.65

^a^Measured using a capillary viscometer [24]

**Fig. 2:**
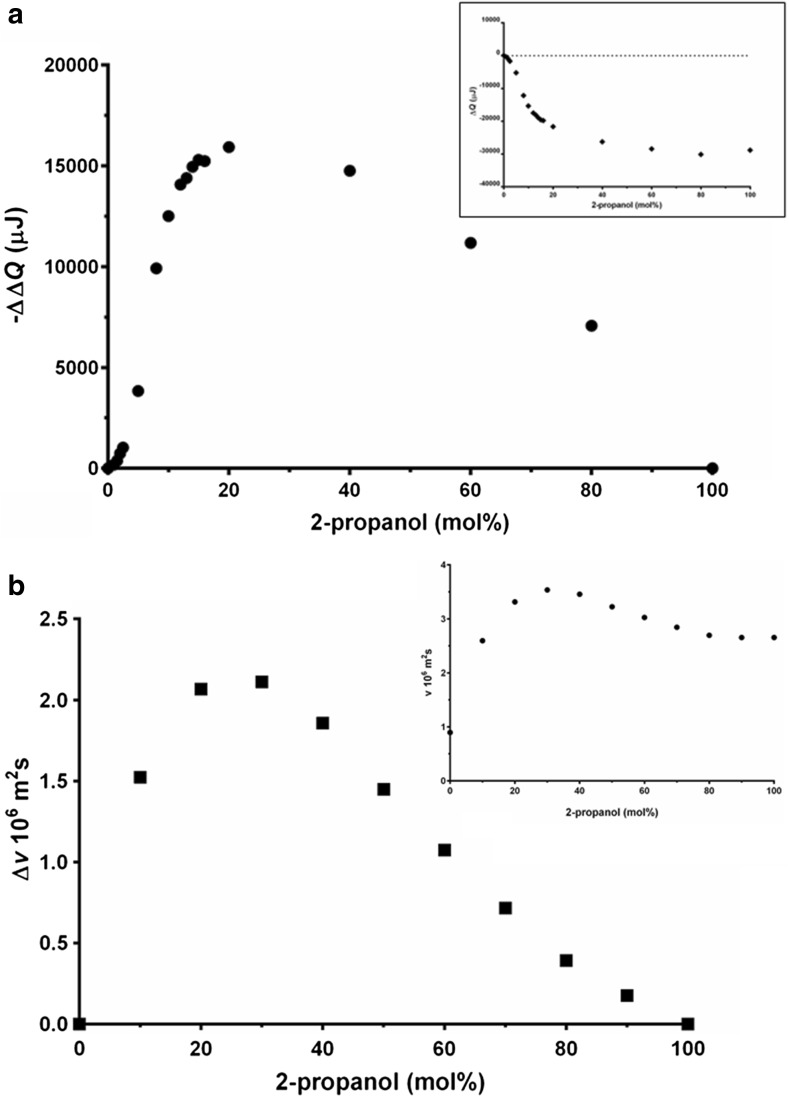
**a** Deviation from linear relationship between heat and 2-propanol concentration at 25.5 °C (the *inset* shows the difference in heat versus 2-propanol concentration at 25.5 °C taken from Fig. 1), and **b** deviation from linear relationship between the viscosity and 2-propanol concentration at 25.0 °C (the *inset* shows the kinematic viscosity vs. 2-propanol concentration at 25.0 °C taken from Soliman and Marschall [24], measured with a capillary viscometer, illustrating the obvious non-linearity in both data set)

When the heat change (Δ*Q*) at 25 °C is plotted against 2-propanol concentration (Fig. 2a, inset) the non-linearity of this relationship is apparent. The difference between the measured Δ*Q* values and those values that would be obtained if the relationship was directly proportional to the molar composition of the solution (ΔΔ*Q*) is plotted as a function of 2-propanol concentration (Fig. 2a). A maximum in ΔΔ*Q* between 20 and 40 mol% 2-propanol is clearly observed. This trend shows a close similarity to the relative kinematic viscosity measurements at 25.5 °C taken from the literature, see Fig. 2b [28]. This is not unexpected as the Δ*Q* value of an aqueous mixture is dominated by the energy released from the hydrogen bonds broken during pressurization. This is related to the viscosity of water where the attractive forces (which in the case of water are dominated by hydrogen bonds) have to break and reform for the molecules to move past each other.

**Fig. 3:**
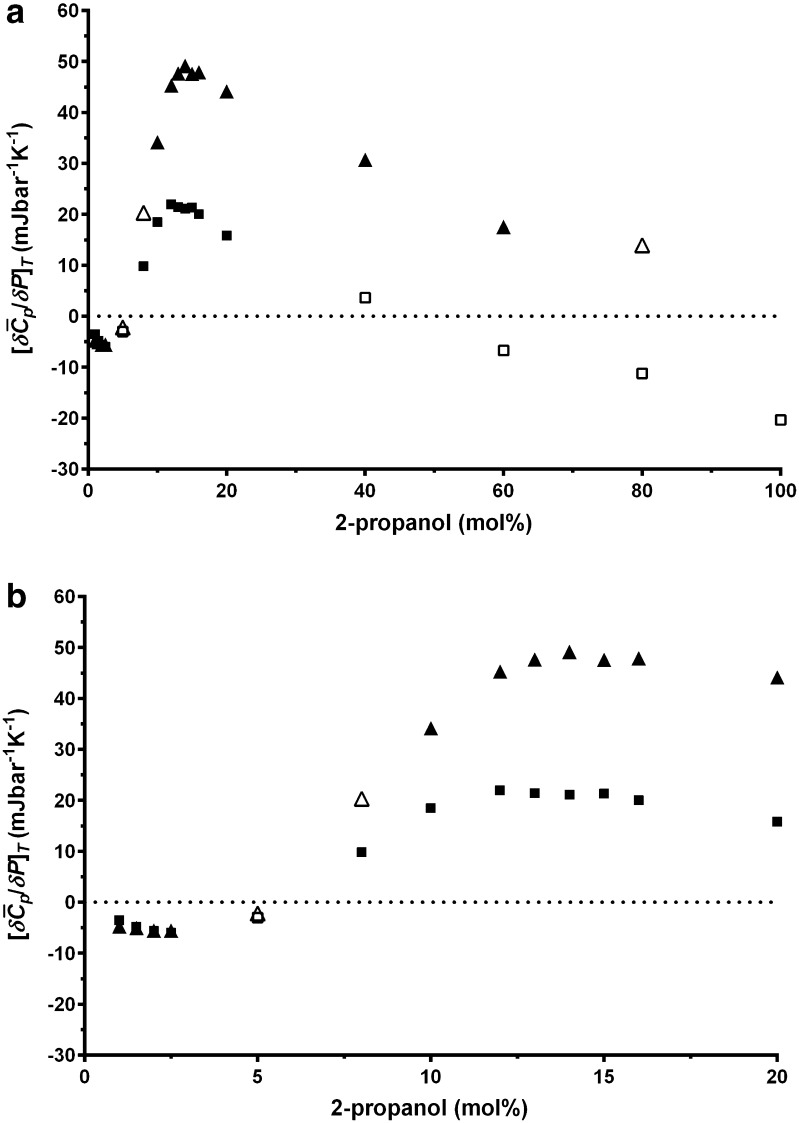
**a** Average gradient $\left[ {\delta \bar{C}_{p} /\delta p} \right]_{T}$ between 9 and 35 °C (*triangles*) or 35 and 61 °C (*squares*) for 2-propanol concentrations between 0 and 100 mol% and **b** between 0 and 20 mol%. The average gradient was calculated using partial least-squares regression. The *filled symbols* have an R^2^ values greater than 0.8 and the *empty symbols* have an R^2^ values less than 0.8

When the calculated average gradients, $\left[ {\delta \bar{C}_{p} /\delta p} \right]_{T} ,$ between 9–35 and 35–61 °C, are plotted against 2-propanol concentration (Fig. 3) a complex relationship is observed. At concentrations between 0 and 2.5 mol%, $\left[ {\delta \bar{C}_{p} /\delta p} \right]_{T}$ is negative and then rises to a peak at 14 ± 2 mol% 2-propanol. The peak at 14 ± 2 mol% 2-propanol is higher in magnitude for the low temperature range (9–35 °C) than for the higher temperature range (35–61 °C). At concentrations above 15 mol% 2-propanol, the value of $\left[ {\delta \bar{C}_{p} /\delta p} \right]_{T}$ decreases with increasing 2-propanol concentration. In the higher temperature range (35–61 °C), $\left[ {\delta \bar{C}_{p} /\delta p} \right]_{T}$ became negative at concentrations between 40 and 60 mol% 2-propanol.

It is noteworthy that these trends in $\left[ {\delta \bar{C}_{p} /\delta p} \right]_{T}$ can be broadly correlated with observations about the Δ*Q* values shown in Fig. 1. Between 1 and 2.5 mol% 2-propanol, Δ*Q* values follow a consistent monotonic trend. Between 5 and 8 mol% they follow an erratic, non-monotonic trend, and then between 10 and 40 mol% the data sets again follow a consistent monotonic trend. Finally, over 40 mol% they became erratic again. This is indicative of the instrument not reaching equilibrium between 5 and 8 mol% and again over 40 mol%. Attempts to overcome this behavior by increasing the time between pressure changes resulted in either very slow equilibration or oscillatory behavior (data not shown).

**Fig. 4:**
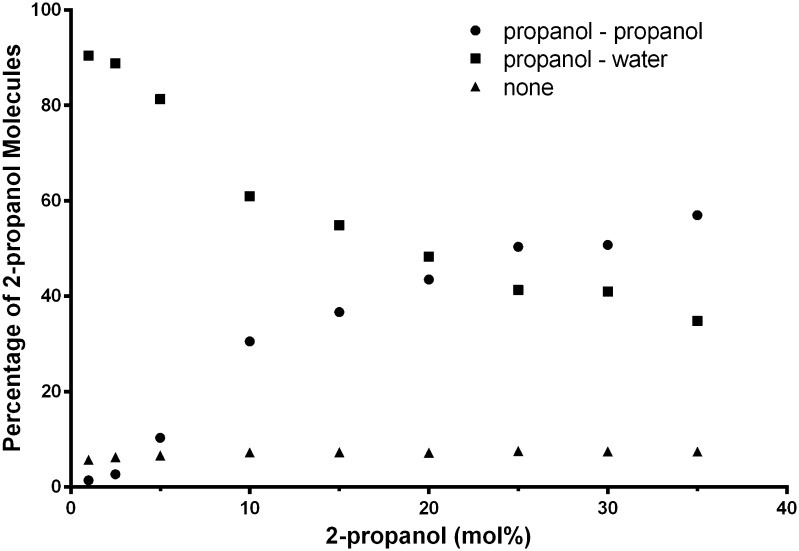
Variation of the percentage of 2-propanol molecules with either hydrogen bonds to other 2-propanol molecules, with water molecules or with no hydrogen bonds, with the concentration of 2-propanol calculated using molecular dynamics simulation

Molecular dynamics (MD) simulation of 2-propanol water mixtures (Fig. 4; Table 2) shows that very few of the 2-propanol molecules are hydrogen bonded to other 2-propanol molecules below a concentration of 2.5 mol% 2-propanol, suggesting that the alcohol molecules exist as isolated species surrounded by water. Above 2.5 mol% 2-propanol the number of hydrogen bonds between 2-propanol molecules steadily rises with a corresponding drop in 2-propanol/water hydrogen bonds, indicating the formation of larger alcohol networks. The rise in hydrogen bonds between 2-propanol molecules slows above 25 mol% 2-propanol. The number of 2-propanol molecules without hydrogen bonds remains constant at approximately 7%.

**Table 2:** Variation of the percentages of 2-propanol molecules with either hydrogen bonds to other 2-propanol molecules (P–P) or to water molecules (P–W), or with no hydrogen bonds, along with the concentration of propanol and the calculated deviations from the random distribution (non-ideality) as determined using molecular dynamic simulation

Concentration of 2-propanol (mol%)	P–P (%)	P–W (%)	No H-bonds (%)	Non-ideality
1	1.42	90.41	5.75	0.076
2.5	2.71	88.83	6.30	0.061
5	10.33	81.29	6.63	0.08
10	30.54	60.95	7.27	0.213
15	36.68	54.9	7.33	0.295
20	43.52	48.31	7.24	0.369
25	50.37	41.3	7.58	0.431
30	50.77	41.02	7.47	0.436
35	57.01	34.84	7.46	0.422

**Fig. 5:**
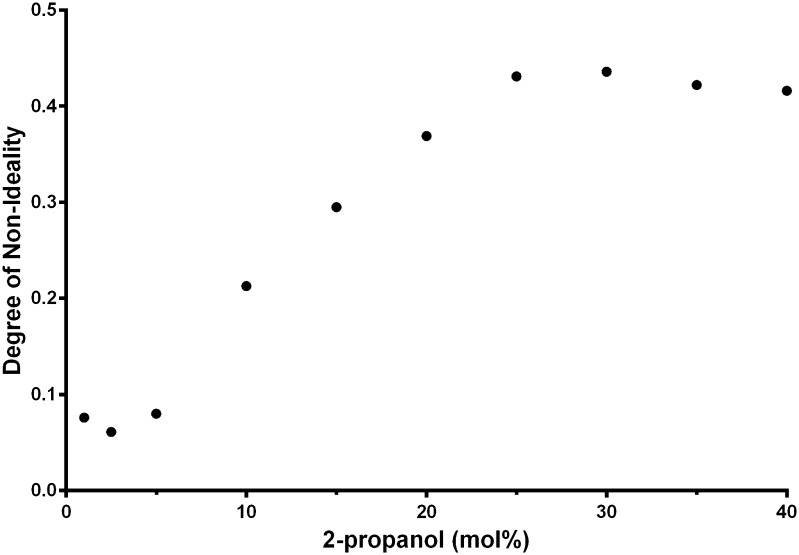
The deviation from the random distribution (non-ideality) versus 2-propanol concentration, calculated using molecular dynamics simulation where non-ideality suggests that segregation of the water and 2-propanol is occurring

The distribution of water and 2-propanol molecules during the course of the MD simulation shows deviations from a random distribution and thereby implies segregation of water and 2-propanol (Fig. 5). Below 2.5 mol% 2-propanol the solution remains effectively a random mixture, suggesting little segregation. Above 2.5 mol% 2-propanol the extent of the deviations from a random distribution rises and reaches a maximum around 25 mol% 2-propanol, indicative of extensive segregation or cluster formation.

## Discussion

A physical interpretation of the Δ*Q* and the $\left[ {\delta \bar{C}_{p} /\delta p} \right]_{T}$ values calculated from the PPC experiments can be postulated through comparison to previous experimental work that provided viscosity [24], excess enthalpy, relative absorbance at 1 THz [25, 26] and activation energy for a molecular diffusive jump of 2-propanol in 2-propanol/water mixtures [25], and to the MD simulation data presented herein.

**Fig. 6:**
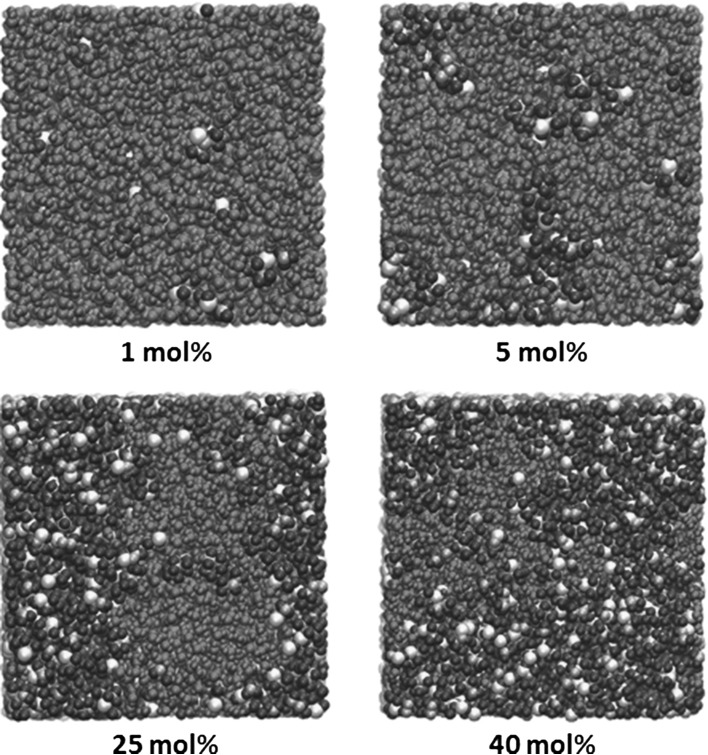
Images of the molecular dynamic simulation of 1, 5, 25 and 40 mol% 2-propanol in water

Below 2.5 mol% 2-propanol the results of the PPC analysis and MD simulation are consistent with individual 2-propanol molecules being surrounded by water molecules with little interaction between the 2-propanol molecules. The image of the MD simulation of 1 mol% 2-propanol illustrates this clearly (Fig. 6). The deviations from random distribution, calculated using MD simulation as the degree of non-ideality, are low between 0 and 5 mol% 2-propanol (Fig. 5), thereby suggesting that the solution is well mixed and little or no segregation has occurred. The drop in Δ*Q* as the 2-propanol concentration rises follows a non-linear trend between 0 and 20 mol% 2-propanol, with the curve more shallow below 2.5 mol% 2-propanol as compared to higher 2-propanol concentrations (Fig. 2). The plot of $\left[ {\delta \bar{C}_{p} /\delta p} \right]_{T}$ versus 2-propanol concentration (Fig. 3) shows a negative value below 5 mol% 2-propanol. This is consistent with previous PPC research where negative values were observed for hydrophobic solutes in water whereas positive values were measured for charged or polar solutes [8].

Between 2.5 and 5 mol% 2-propanol there is a transition where segregation of 2-propanol and water molecules starts to occur. This is most clearly observed in the $\left[ {\delta \bar{C}_{p} /\delta p} \right]_{T}$ versus 2-propanol concentration plot (Fig. 3) where the $\left[ {\delta \bar{C}_{p} /\delta p} \right]_{T}$ value starts to rise and becomes positive at a concentration just over 5 mol% 2-propanol. The drop in the Δ*Q* values also becomes steeper at this point. At 2-propanol concentrations greater than 2.5 mol% the 2-propanol molecules therefore start to interact with each other and isolated 2-propanol molecules with discrete hydration layers are no longer present. This is captured by MD simulation (Figs. 4, 5) as a rise in hydrogen bonding between 2-propanol molecules and deviation from a random distribution of the mixture. The Δ∆*Q* and the $\left[ {\delta \bar{C}_{p} /\delta p} \right]_{T}$ values measured by PPC also rise (Figs. 2, 3) as does kinematic viscosity [22], excess enthalpy, relative absorbance at 1 THz [25, 26], and activation energy for a molecular diffusive jump of 2-propanol in 2-propanol water mixtures [25]. The peaks in these values, however, are not consistent. The excess enthalpy and the peak in activation energy for a molecular diffusive jump of 2-propanol in 2-propanol–water mixtures occur around 10 mol% 2-propanol [25]. The $\left[ {\delta \bar{C}_{p} /\delta p} \right]_{T}$ values and relative absorbance at the 1 THz [25, 26] peak is between 12 and 18 mol% 2-propanol.

Previous THz-TDS analysis suggested that below 2 mol% 2-propanol, 2-propanol exists as discrete molecules with an extensive hydration layer surrounding each alcohol molecule [26]. Above 2 mol% the 2-propanol molecules start to interact with each other [26]. THz-TDS measured a maximum in absorption relative to an ideal solution at around 10–15 mol% 2-propanol while the relative relaxation strength of 2-propanol, as measured by NMR, also has a maximum between 10 and 15 mol% 2-propanol [25, 26]. Neutron diffraction at two 2-propanol concentrations (10 and 30 mol%) indicated that there was a heterogeneous mixture of interactions between 2-propanol and water [25]. At 10 mol% 2-propanol there is significant clustering of 2-propanol, as evidenced by the number of hydrogen bonds between alcohol molecules; however, at 30 mol% 2-propanol there are increased 2-propanol–water interactions indicating a relatively well mixed solution. These results were interpreted as evidence that the maximum extent of the alcohol water network occurs at around 10 mol% 2-propanol.

The kinematic viscosity [24] and the −Δ∆*Q* peak are between 20–30 mol% 2-propanol which does not match the peak at 14 ± 2 mol% 2-propanol detected by $\left[ {\delta \bar{C}_{p} /\delta p} \right]_{T} ,$ THz spectroscopy, NMR relaxation and neutron diffraction studies [25]. The Δ∆*Q* and kinematic viscosity peak between 20–30 mol% 2-propanol coincides with the maximum in degree of non-ideality determined by the MD simulation and suggests this peak is not directly related to the 2-propanol/water hydrogen bonded network detected by $\left[ {\delta \bar{C}_{p} /\delta p} \right]_{T}$ and THz-TDS [25]. This does suggest a complex relationship between water and 2-propanol within mesoscopic structured mixtures. The properties of the binary mixtures that exhibit peaks between 10 and 30 mol% all have the same ultimate physical origin, i.e. the breaking and reforming of hydrogen bonds, but are different manifestations of this, involving distinct physical processes and hence there is no precise overlap between the values.

At higher concentrations of 2-propanol (greater than 20–30 mol%) the further addition of 2-propanol can disrupt the 2-propanol/water networks; however the difference in $\left[ {\delta \bar{C}_{p} /\delta p} \right]_{T}$ between 9–21 and 49–61 °C suggests that in the lower temperature regime the 2-propanol/water hydrogen bonded networks are more extensive and persistent. They do not breakdown readily with addition of more 2-propanol, this was observed as a stable positive $\left[ {\delta \bar{C}_{p} /\delta p} \right]_{T}$ value. At 49–61 °C the 2-propanol/water hydrogen bonded networks are less stable and readily break down with addition of 2-propanol, this is observed as a decline in $\left[ {\delta \bar{C}_{p} /\delta p} \right]_{T} .$

The Δ*Q* value of the 2-propanol water mixtures is, by the nature of the experiment, relative to ultra-pure water and is dominated by the energy released from the hydrogen bonds broken during pressurization. This is related to viscosity of water where the attractive forces (in the case of water dominated by the hydrogen bonds) have to break and reform for the molecules to move past each other. We therefore contend that the similarity of the Δ*Q* value (Fig. 2a) and kinematic viscosity (Fig. 2b) is not coincidental as both arise from the same mechanistic origin.

## Conclusion

The application of PPC to the analysis of 2-propanol/water mixtures has, through comparison to complementary molecular dynamic simulations, been shown to yield informative data. The change in heat, Δ*Q*, is indicative of bond breaking upon pressurization and is therefore correlated with viscosity and diffusivity. The $\left[ {\delta \bar{C}_{p} /\delta p} \right]_{T}$ values are related to the mesoscopic structure of the 2-propanol/water mixture. PPC data suggest the hydration layers around individual 2-propanol molecules (at <2.5 mol% 2-propanol) can be detected by the negative $\left[ {\delta \bar{C}_{p} /\delta p} \right]_{T}$ values relative to pure water. PPC can also detect the stable, constrained 2-propanol/water networks (with a peak at 14 ± 2 mol% 2-propanol) which are clearly detected as a positive peak in $\left[ {\delta \bar{C}_{p} /\delta p} \right]_{T}$ relative to pure water. PPC is a relatively straightforward analytical technique that supplies useful data for solvent–solvent and solvent–solute mixtures. It takes less than one day to acquire a complete data set for a sample covering a wide temperature range (7–62 °C), has a low operational cost and uses readily available commercial microcalorimetry DSC instruments, making it practical for routine laboratory use, and hence represents a valuable addition to the experimental toolkit in this field.

## Electronic supplementary material

Below is the link to the electronic supplementary material.

Supplementary material 1 (DOCX 391 kb)
