# Supplementary material for: Analysis of Mesoscopic Structured 2-Propanol/Water Mixtures Using Pressure Perturbation Calorimetry and Molecular Dynamic Simulation
Source: J Solution Chem. 2016 Dec 17;46(1):175–89. doi: 10.1007/s10953-016-0554-y (PMC5241351; doi:10.1007/s10953-016-0554-y)
Supplement: Supplementary file 1 — Supplementary material 1 (DOCX 391 kb) [file 10953_2016_554_MOESM1_ESM.docx]

**Supplementary Information for:**

**Analysis of Mesoscopic Structured 2-Propanol/Water Mixtures using Pressure Perturbation Calorimetry and Molecular Dynamic Simulation**

**Jordan W. Bye^1^ · Colin L. Freeman^2^ · John D. Howard^1^ · Gregor Herz^1^ · James McGregor^1^ · Robert J. Falconer^1^**

^1^ Department of Chemical & Biological Engineering, ChELSI Institute, University of Sheffield, Sheffield, S1 3JD, England

^2^ Department of Material Science & Engineering, University of Sheffield, Sheffield, S1 3JD, England

**S.1 Plots of Raw Pressure Perturbation Calorimetry Data**





**Fig. S1** Raw data from a pressure perturbation scan of 2.5 mol% isopropanol in water. Alternating pressure pulses from 1→5 bar and then 5→1 bar were applied to the sample at 1 °C intervals from 7 °C to 56 °C, with a heating rate of 0.1 °C·min^–1^



**Fig. S2** Raw data from a pressure perturbation scan of 5 mol% isopropanol in water. Alternating pressure pulses from 1→5 bar and then 5→1 bar were applied to the sample at 1 °C intervals from 7 °C to 56 °C, with a heating rate of 0.1 °C·min^–1^



**Fig. S3** Raw data from a pressure perturbation scan of 15 mol% isopropanol in water. Alternating pressure pulses from 1→5 bar and then 5→1 bar were applied to the sample at 1 °C intervals from 7 °C to 56 °C, with a heating rate of 0.1 °C·min^–1^



**Fig. S4** Close up of Fig. S3 from 20 °C to 25 °C, with data for pressure increases and decreases shown (---)

**S2 Calculation of** $\left[ {{\boldsymbol{\delta}\overline{\boldsymbol{C}}}_{\boldsymbol{p}}}/\boldsymbol{\delta p} \right]_{\boldsymbol{T}}$

The change in pressure was 4 bar, the change in temperature was 2 °C, the volume of the sample was 300 μL and the DSC measured the heat required to maintain the temperature of the sample. The $\left[ {{\delta\overline{C}}_{p}}/{\delta p} \right]_{T}$ value used in the paper is for 1 L of the 2-propanol water mixture.

**S3 Derivation of the Equations used for Data Analysis of Pressure Perturbation Calorimetry Output**

**S3.1 Lin et al.’s PPC Equations**

The equations used by Lin et al 2002 to explain PPC data were based on the second law of thermodynamics as the starting point [1]:

$dS={{dQ}_{\mathrm{rev}}}/T$ (S1)

where *dS* is the entropy change, *dQ*_rev_ is the heat change for a reversible process and *T* is temperature. Differentiating with respect to pressure at constant temperature gives

$\left( {{\partial Q}_{\mathrm{rev}}}/{\partial p} \right)_{T}={T\left( {\partial S}/{\partial p} \right)}_{T}$ (S2)

Maxwell’s relation is

$\left( {\partial S}/{\partial p} \right)_{T}={-\left( {\partial V}/{\partial T} \right)}_{p}$ (S3)

Substitute into Eq. S2 gives

$\left( {{\partial Q}_{\mathrm{rev}}}/{\partial p} \right)_{T}={-T\left( {\partial V}/{\partial T} \right)}_{p}=-TV\alpha$ (S4)

where *V* is the volume, *p* is the pressure and *α* is coefficient of thermal expansion. Integration of Eq. S4 at constant temperature and a small change in the pressure (assuming *V* and *α* are constant with small pressure changes) gives:

$Q_{\mathrm{rev}}=-TV\alpha\Delta p$ (S5)

For a two-component solution

$V_{\mathrm{total}}=g_{0}V_{0}+g_{s}\overline{V}$ (S6)

where *V*_total_ is total solution volume, *V*_0_ is specific volume of the solvent, $\overline{V}$ is the partial specific volume of the solute, *g*_0_ is weight of the solvent and *g_s_* is weight of the solute. Differentiation of Eq. S6 with respect to temperature while at constant pressure gives

$\left( {\partial V_{\mathrm{total}}}/{\partial T} \right)_{p}=g_{0}\left( {\partial V}/{\partial T} \right)_{p}+g_{s}\left( {\partial\overline{V}}/{\partial T} \right)_{p}$ (S7)

Substituting Eq. S7 into Eq. S4 gives

$\left( {{\partial Q}_{\mathrm{rev}}}/{\partial p} \right)_{T}=-T\left[ g_{0}\left( {\partial V}/{\partial T} \right)_{p}+g_{s}\left( {\partial\overline{V}}/{\partial T} \right)_{p} \right]$ (S8)

Inasmuch as *α* = 1/*V*$\left( {\partial V}/{\partial T} \right)_{p}$, Eq. S8 gives

$\left( {{\partial Q}_{\mathrm{rev}}}/{\partial p} \right)_{T}=-T\left[ g_{0}V_{0}\alpha_{0}+g_{s}\overline{V}\overline{\alpha} \right]$ (S9)

where *α*_0_ is the coefficient of thermal expansion of the solvent, $\overline{\alpha}$ is the coefficient of thermal expansion of the solute’s partial volume. Integration of Eq. S9 (assuming a small pressure change) gives

$Q_{\mathrm{rev}}=-T\left[ g_{0}V_{0}\alpha_{0}+g_{s}\overline{V}\overline{\alpha} \right]\Delta p$ (S10)

During PPC operation there are two cells (a reference containing solvent only and a sample cell containing solvent and solute) with identical volumes and identical pressure change so the net heat is the difference given by Eq. S10 for the two cells. The difference arises from the volume occupied by the solute in the sample cell, so

${\Delta Q}_{\mathrm{rev}}=-T\Delta P\left[ g_{s}\overline{V}\overline{\alpha}-{g_{s}\overline{V}\alpha}_{0} \right]$ (S11)

Rearranged this equation gives

${\Delta Q_{\mathrm{rev}}}/{T\Delta P}=\left( \alpha_{0}-\overline{\alpha} \right)\overline{V}g_{s}$ (S12)

Note: this derivation was created by the authors of Lin et al. [1] who built on the work of Kujawa and Winnik [2]. An expression similar to Eq. S12 was previously used by Ter Minassian and Pruzan in 1977 [3].

Comment: The partial specific volume of the solute ($\overline{V}$) is comprised of the volume of the solute plus the change in volume of the solvent caused by interactions with the solute. A core assumption of the equations derived by Lin et al. is that ($\overline{V}$) is constant irrespective of the solute’s concentration. In the 1940s, when much of the original work on partial specific volumes was undertaken, the partial molal volume was obtained by extrapolating the apparent specific volume to infinite dilution. The partial specific volume of solutes were expressed as being at infinite dilution due to the possibility of ion pair interactions at higher concentrations or due to the change in volume of the solvent caused by interactions with the solute that vary with solute concentration. The PPC work undertook by Bye and Falconer showed non-linearity in the ∆*Q* versus salt concentration plot, suggesting that the molar expansivity (*E* = *αV*) was affected by solute concentration [4].

**S3.2 Falconer’s PPC Equations**

Bye and Falconer [4] used a three-component model to avoid the ambiguities associated with the use of the partial specific volume of the solute. With the advent of molecular dynamic simulation it became possible to estimate the number of water molecules that are present around small solutes like ion pairs. Their equations to explain PPC data, Eq. S5, was taken from Lin et al. [1]:

$V_{\mathrm{tot}}=x_{b}V_{b}+x_{h}V_{h}+x_{s}V_{s}$ (S13)

where *V*_b_ is the molar volume of bulk solvent, *V*_h_ is the average molar volume of the solvent within the hydration layer and *V*_s_ is the molar volume of solute. Also, *x*_b_ is the molar fraction of the bulk solvent, *x*_h_ is the molar fraction of the solvent within the hydration layer and *x*_s_ is the molar fraction of the solute.

For a three component system Eq. S5 becomes

$Q=-T\Delta p\left[ x_{b}V_{b}\alpha_{b}+x_{h}V_{h}\alpha_{h}+x_{s}V_{s}\alpha_{s} \right]$ (S14)

where *α*_b_, *α*_h_ and *α*_s_ are the thermal expansion coefficient of the bulk water, hydration layer and solute, respectively.

The difference in heat between the sample and reference cells is

$\Delta Q=T\Delta p\left[ {x_{0}V_{0}\alpha_{0}-x}_{b}V_{b}\alpha_{b}-x_{h}V_{h}\alpha_{h}-x_{s}V_{s}\alpha_{s} \right]$ (S15)

where x_0_, *V*_0_ and *α*_0_ are the molar fraction, molar volume and thermal expansion coefficient of the pure water in the reference cell. As *V*_0_ = *V*_b_, *α*_0_ = *α*_b_ and *x*_0_ = 1 the equation can be simplified to

$\Delta Q=T\Delta p\left[ \left( {1 -x}_{b} \right)\left( V_{b}\alpha_{b} \right)-x_{h}V_{h}\alpha_{h}-x_{s}V_{s}\alpha_{s} \right]$ (S16)

As *x_b_* = 1 – *x_h_* – *x*_s_,

$\Delta Q=T\Delta p\left[ \left( {x_{s}+x}_{h} \right)\left( V_{b}\alpha_{b} \right)-x_{h}V_{h}\alpha_{h}-x_{s}V_{s}\alpha_{s} \right]$ (S17)

When *x*_h_ is defined by the multiple number (*n*) of water molecules around the ions *x*_h_ = *nx*_s_, the equation can be further simplified to

$\Delta Q=T\Delta p\left[ \left( n+1 \right)\left( x_{s}V_{b}\alpha_{b} \right)-{nx}_{s}V_{h}\alpha_{h}-x_{s}V_{s}\alpha_{s} \right]$ (S18)

$\Delta Q=T\Delta px_{s}\left[ \left( n+1 \right)\left( V_{b}\alpha_{b} \right)-nV_{h}\alpha_{h}-V_{s}\alpha_{s} \right]$ (S19)

The experimental results show that as Δ*p* and *x*_s_ tend to zero Δ*Q* tends to zero but as *T* tends to zero Δ*Q* becomes negative; therefore, a constant (*A*) is added to the equation. Note *A* is undefined and may be due to the presence of the sodium cation and its interaction with the anion. Here *T* is the variable in:

$\Delta Q=T\Delta px_{s}\left[ \left( n+1 \right)\left( V_{b}\alpha_{b} \right)-nV_{h}\alpha_{h}-V_{s}\alpha_{s} \right]+A$ (S20)

Note, this derivation is that of Bye and Falconer [4] who built on the work of Lin et al. [1].

Comment: this mathematics relies on the assumption of discrete solute molecules with hydration layers that do not overlap. A criticism of this mathematics and that of Brandt can be made for 2-propanol water mixtures where segregation can occur at concentrations over 2.5 mol%.

Comment: the core assumption in this work is that the hydration layer around an anion and cation can be restricted to a set number of water molecules (*n*). The setting of the limit to the hydration layer may be restricted to a single layer of water [5], but there is a case for extending it to a second layer [6]. It is worth noting that $V_{h}\alpha_{h}$ will be an average for the water molecules within the hydration layer.

**S 3.3The use of PPC Equations to Interpret 2-Propanol Water Mixture Data**

While the use of Eqs. 12 or 20 could be justified for 2-propanol water mixtures below 2.5 mol% they certainly don’t explain the interactions at concentrations of 2-propanol above 2.5 mol%.

Equation S5 (${Q_{\mathrm{rev}}}/{T\Delta p}=-V\alpha$) does suggest that the net expansivity of the 2-propanol water mixture is closely related to the ${Q_{\mathrm{rev}}}/{T\Delta p}$ value and that the PPC data, where the sample cell with the 2-propanol water mixture is run in parallel with a pure water reference, does indicate that a decrease in net molar expansivity can deduced from a rise in ${Q_{\mathrm{rev}}}/{T\Delta p}$.

**References**

1. Lin, L.N., Brandts, J.F., Brandts, J.M., Plotnikov, V.: Determination of the volumetric properties of proteins and other solutes using pressure perturbation calorimetry. Anal. Biochem. **302**, 144–60 (2002)

2. Kujawa, P., Winnik, F.M.: Volumetric studies of aqueous polymer solutions using pressure perturbation calorimetry: A new look at the temperature-induced phase transition of poly(N-isopropylacrylamide) in water and D_2_O. Macromolecules **34**, 4130–4135 (2001)

3. Ter Minassian, L., Pruzan, P.: High-pressure expansivity of materials determined by piezo-thermal analysis. J. Chem. Thermodyn. **9**, 375–390 (1977)

4. Bye, J.W., Falconer, R.J.: A study of the relationship between water and anions of the Hofmeister series using pressure perturbation calorimetry. Phys. Chem. Chem. Phys. **17**, 14130–14137 (2015)

5. Bakker, H.J.: Structural dynamics of aqueous salt solutions. Chem. Rev. **108**, 1456–1473 (2008)

6. Mancinelli, R., Botti, A., Bruni, F., Ricci, M.A., Soper, A.K.: Perturbation of water structure due to monovalent ions in solution. Phys. Chem. Chem. Phys. **9**, 2959–2967 (2006)
